# Effect of Irrigation Fluid Temperature on Recurrence in the Evacuation of Chronic Subdural Hematoma: A Randomized Clinical Trial

**DOI:** 10.1001/jamaneurol.2022.4133

**Published:** 2022-11-21

**Authors:** Andreas Bartley, Jiri Bartek, Asgeir S. Jakola, Jimmy Sundblom, Marie Fält, Petter Förander, Niklas Marklund, Magnus Tisell

**Affiliations:** 1 Institute of Neuroscience and Physiology, Department of Clinical Neuroscience, University of Gothenburg, Sahlgrenska Academy, Gothenburg, Sweden; 2 Department of Neurosurgery, Sahlgrenska University Hospital, Gothenburg, Sweden; 3 Department of Neurosurgery, Karolinska University Hospital, Stockholm, Sweden; 4 Department of Clinical Neuroscience, Karolinska Institutet, Stockholm, Sweden; 5 Department of Neurosurgery, Copenhagen University Hospital Rigshospitalet, Copenhagen, Denmark; 6 Department of Medical Sciences; Neurosurgery, Uppsala University, Uppsala, Sweden; 7 Department of Clinical Sciences Lund, Neurosurgery, Lund University, Skåne University Hospital, Lund, Sweden

## Abstract

**Importance:**

The effect of a physical property of irrigation fluid (at body vs room temperature) on recurrence rate in the evacuation of chronic subdural hematoma (cSDH) needs further study.

**Objective:**

To explore whether irrigation fluid temperature has an influence on cSDH recurrence.

**Design, Setting, and Participants:**

This was a multicenter randomized clinical trial performed between March 16, 2016, and May 30, 2020. The follow-up period was 6 months. The study was conducted at 3 neurosurgical departments in Sweden. All patients older than 18 years undergoing cSDH evacuation during the study period were screened for eligibility in the study.

**Interventions:**

The study participants were randomly assigned by 1:1 block randomization to the cSDH evacuation procedure with irrigation fluid at room temperature (RT group) or at body temperature (BT group).

**Main Outcomes and Measures:**

The primary end point was recurrence requiring reoperation within 6 months. Secondary end points were mortality, health-related quality of life, and complication frequency.

**Results:**

At 6 months after surgery, 541 patients (mean [SD] age, 75.8 [9.8] years; 395 men [73%]) had a complete follow-up according to protocol. There were 39 of 277 recurrences (14%) requiring reoperation in the RT group, compared with 16 of 264 recurrences (6%) in the BT group (odds ratio, 2.56; 95% CI, 1.38-4.66; *P* < .001). There were no significant differences in mortality, health-related quality of life, or complication frequency.

**Conclusions and Relevance:**

In this study, irrigation at body temperature was superior to irrigation at room temperature in terms of fewer recurrences. This is a simple, safe, and readily available technique to optimize outcome in patients with cSDH. When irrigation is used in cSDH surgery, irrigation fluid at body temperature should be considered standard of care.

**Trial Registration:**

ClincalTrials.gov Identifier: NCT02757235.

## Key Points

**Question:**

Surgery for a chronic subdural hematoma is one of the most common neurosurgical procedures performed, but recurrence rates are still high; evidence-based guidelines for optimal management are needed to reduce recurrence.

**Findings:**

In this randomized clinical trial of 541 individuals undergoing evacuation of chronic subdural hematoma, results showed that irrigation fluid at body temperature leads to fewer hematoma recurrences compared with irrigation fluid maintained at room temperature.

**Meaning:**

This study explored how a physical property, such as irrigation fluid temperature, can affect outcomes of neurosurgery for chronic subdural hematoma.

## Teaser

This randomized clinical trial investigates the effect of irrigation fluid temperature on hematoma recurrence in the evacuation of chronic subdural hematoma.

## Introduction

A chronic subdural hematoma (cSDH) consists of a collection of old blood and degraded blood products located in the subdural space. A process driven by inflammatory and angiogenetic factors may cause the subdural collection of blood to progress to a significant volume causing symptoms due to compression of the brain.^1^ Common symptoms of cSDH are focal neurologic deficits, an altered mental state, and symptoms related to increased intracranial pressure, such as headache, decreased consciousness, and in severe cases, even death. Older adults (>65 years) are especially susceptible to cSDH due to a higher frequency of anticoagulant therapy, brain atrophy, and an increased risk of head trauma due to falls.^2^ The increased cSDH incidence is largely attributed to an aging population in combination with increased use of antithrombotic therapy.^3^ Data from the Nordic countries indicate that the incidence has almost tripled, and the number of surgeries doubled, in the last 20 to 30 years.^4,5^ These findings are consistent with an increasing incidence of cSDH in the US, where the annual case load is predicted to be 60 000 cases by 2030.^6^

The most common surgical technique is evacuation via 1 to 2 cranial burr holes combined with the use of a postoperative drain.^7^ It is common practice to irrigate the subdural space during surgery.^8,9^ Still, recurrences of cSDH requiring reoperation are in the range of 10% to 20%.^10^ Recurrence is associated with increased morbidity and mortality.^11,12^A significant reduction of recurrence rates would be beneficial for both individual patients and health care spending.

In theory, irrigation fluid temperature may influence recurrence rates after cSDH surgery. The increased solubility with irrigation fluid at body temperature compared with room temperature may facilitate cSDH clearance.^13^ There may also be negative effects on coagulation when using fluid at room temperature.^14^ Despite these theoretical benefits of irrigation at body temperature, it is still common to use irrigation fluid at room temperature. An internet poll performed in 2017 showed that of 620 responding neurosurgeons, 57% used irrigation at body temperature, 40% used irrigation at room temperature, and 3% did not use irrigation at all.^15^ To investigate this, we performed a pilot study, where recurrence in need of reoperation was reduced from 13.4% to 4.5% following a switch to irrigation fluid set to body temperature.^16^ In that single-center retrospective study, there was a clear risk of a Hawthorne effect (ie, observation changes behavior) due to the focus on a temporary change in clinical practice. To clarify this, we initiated this randomized clinical trial. Our primary aim with this study was to compare the effect of irrigation fluid at body temperature (BT group) vs room temperature (RT group) on the risk of recurrence after cSDH surgery within a 6-month follow-up period. Secondary aims were a comparison between the 2 groups regarding mortality, complication frequency, and health-related quality of life at 6 months.

## Methods

### Trial Design and Oversight

This was a multicenter randomized clinical trial with parallel-group design (1:1). Patients were stratified according to treating institution. We evaluated the intraoperative use of irrigation fluid at body temperature (37 °C, BT group) vs room temperature (22 °C, RT group) in burr hole evacuation of cSDH. The study protocol has previously been published in addition to the registration at ClinicalTrials.org (Supplement 1).^15^ Study site investigators and locations are listed in the eAppendix of Supplement 2. The study has been prepared in accordance with the Consolidated Standards of Reporting Trials (CONSORT) reporting guidelines and was conducted at 3 Swedish institutions (Gothenburg, Uppsala, and Stockholm) between March 16, 2016, and May 30, 2020.^17^ Each institution has a population-based catchment area. Combined, the participating institutions cover approximately 60% of the Swedish population. Ethical approval was obtained from the Regional Ethics Committee in Gothenburg, Sweden, on January 19, 2016. Except for the randomized treatment allocation, the management did not differ from the institutional standard of care. Burr hole evacuation with intraoperative irrigation combined with a postoperative subgaleal drain was used in all centers.^18,19^ General or local anesthesia was used, and 1 to 2 adjacent burr holes were placed over the cSDH. Ringer lactate at either body or room temperature, depending on the randomization, was used for the irrigation. A soft catheter and a syringe with the irrigation fluid were used to irrigate the subdural space. To avoid inadvertent cooling of the body-tempered fluid, the irrigation fluid was kept in a heating cabinet until use. Following irrigation, a subgaleal drain was inserted over the burr hole and tunneled away from the skin incision. The drain was connected to a collection bag with active suction. The patient was kept in the supine position until drain removal, usually the day after surgery. Variables during the hospital admission were recorded in a case report form preoperatively, intraoperatively, and within 24 hours postoperatively by the treating neurosurgeon. The preoperative computed tomography (CT) scans were reviewed by the treating neurosurgeon, and hematomas were classified as hypodense, isodense, hyperdense, or membranous. Combinations of these CT classifications could occur. A detailed description of all variables recorded can be found in the study protocol (Supplement 1).^15^

### Trial Participants

All patients with a cSDH where surgical treatment was indicated were screened for inclusion in the study. Inclusion criteria were (1) cSDH requiring burr hole evacuation and (2) patients older than 18 years. Exclusion criteria were as follows: (1) surgical treatment for cSDH by means other than burr hole evacuation, (2) patients with a cerebrospinal fluid shunt, (3) patients with intracranial arachnoidal cysts, and (4) patients who have undergone intracranial surgery previously. Study participants with bilateral cSDH were treated with the same treatment allocation on both sides and included as a single study participant. Written informed consent was obtained from either participants or in cases where not possible, the documented next of kin. Race and ethnicity information is not routinely recorded in Sweden; thus, it was not collected for this study.

### Randomization and Blinding

A total of 600 opaque envelopes (200 for each site, block randomized 1:1) with sequential study numbers containing randomly assigned irrigation fluid temperatures were prepared by an independent statistician. The envelopes were kept sealed at each site until the included patient was draped in the operation room. The envelope was opened by the neurosurgeon performing the cSDH surgery. It was not possible to blind the treating surgeon to the treatment allocation. To minimize bias, the patient was not informed of treatment allocation, and the treatment allocation was not recorded in the medical records as this could bias the indication for reoperation. An independent statistician decoded the individual treatment allocations first at the completion of the study, thus making sure that the investigator performing the final statistical analyses was blinded to treatment allocation until the statistical analyses were completed.

### End Points and Assessments

The primary end point was recurrence in need of reoperation within 6 months after index surgery. Recurrence was defined as reoperation of same-sided cSDH, and in cases of bilateral hematomas, a unilateral recurrence would suffice to be recorded as a recurrence. Secondary end points were proportions of complications leading to hospital admission within 30 days, mortality within 6 months, and quality of life measured by the European Quality of Life, 5 dimensions, 3-level version (EQ-5D-3L) after 6 months. The EQ-5D-3L score at 6 months was presented as a utility index score with results ranging from 1 (perfect health) to −0.62 (health state worse than death).^20^At 2 months (or earlier if the study participants developed symptoms consistent with recurrent cSDH), a CT scan was recommended to assess for any residual hematoma. Of note, we did not routinely perform early postoperative CT scans. Usual clinical practice was used as an indication for reoperation and was a decision made by the on-call neurosurgical team, usually by a combination of recurrent or persistent symptoms and a CT scan showing cSDH with mass effect.

### Statistical Analysis

Analysis was performed according to the intention-to-treat principle. The intention-to-treat population consisted of all randomly assigned study participants except for those lost to follow-up and those who withdrew their consent. The primary end point (recurrence) was analyzed by the χ^2^ test for frequency comparison. Secondary end points were analyzed depending on the type of data. Categorical data was compared with χ^2^ test. Normally distributed numerical data was analyzed by *t* test and the Mann-Whitney *U* test if skewed. Normality of data was tested with Shapiro Wilks. A 2-sided *P* value of <.05 was considered statistically significant. The power calculations of sample size were based on the results obtained from our previously published retrospective study.^16^ The total number of patients needed were 496 for a power of 80%. To compensate for attrition in this frail cohort, we decided to prepare a total of 600 randomized treatment allocations. A 1:1 block randomization was performed with stratification for institution. All new inclusions were stopped after the intended sample size was reached at 6 months. Data were analyzed using SPSS software, version 25 (IBM), and Excel, version 2202 (Microsoft).

## Results

### Study Participants

**Figure 1.**
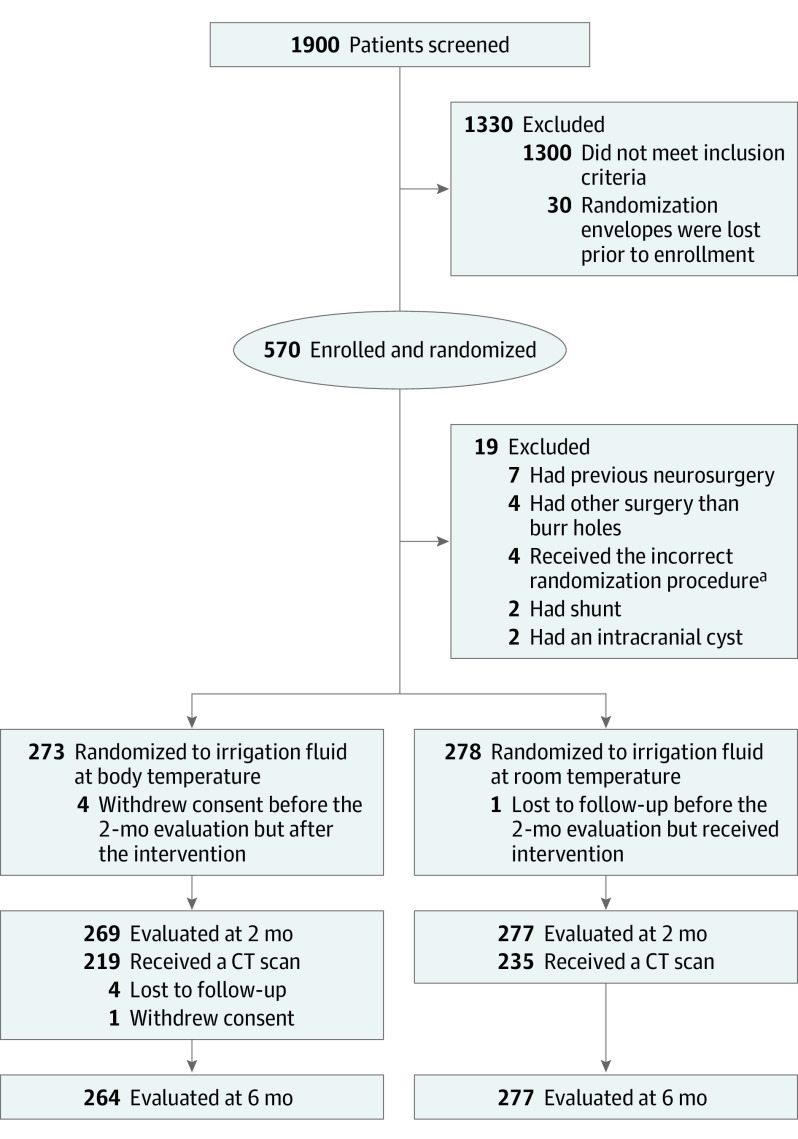
Enrollment, Randomization, and Follow-up ^a^Of 19 patients who were excluded immediately, 4 had an incorrect randomization procedure. This refers to 4 patients with bilateral chronic subdural hematoma undergoing surgery in 2 sessions and randomization envelopes were erroneously used for both sessions. All 4 patients were treated bilaterally according to their first randomization allocation. CT indicates computed tomography.

During the study period approximately 1900 patients were screened for eligibility. In accordance with our study protocol, we did not register the reasons why patients were not enrolled in the study. Patient inclusion and randomization are outlined in Figure 1. We enrolled 570 patients in the study, of whom 19 patients were excluded immediately after randomization due to ineligibility regarding inclusion criteria or an incorrect randomization procedure. Five participants withdrew their consent, and 5 were lost during the follow-up period (2 were not Swedish citizens, and 3 moved abroad during the study). Finally, 277 patients in the RT group and 264 in the BT group for a total of 541 patients (mean [SD] age, 75.8 [9.8] years; 395 men [73%]; 146 women [27%]) had a complete follow-up.

The Table shows the preoperative and intraoperative characteristics of both study groups. With the exception for disorientation/confusion at admission, the treatment groups were balanced. The groups did not significantly differ with respect to age, sex, presenting symptoms, intraoperative variables, antithrombotic therapy, hematoma size, midline shift, or the radiological appearance on CT at baseline (membranous, isodense, hypodense, hyperdense). A first postoperative clinical examination was made within 24 hours, typically the morning after surgery. The were no statistically significant differences between groups with respect to the postoperative clinical examination. Overall, a complete regression or improvement of symptoms was seen in more than 95% of the study participants in both groups. An overview of the postoperative clinical examination results within 24 hours is available in eTable 1 in Supplement 2.

**Table.** Characteristics of the Patients Preoperatively and Intraoperatively

Variable	No./total No. (%)	
	BT group (n = 264)^a^	RT group (n = 277)
Age, mean (SD), y	75.4 (10.1)	76.2 (9.55)
Women	74/264 (28)	72/277 (26)
Men	190/264 (72)	205/277 (74)
Symptoms at admission^b^		
Glasgow Coma Scale score^c^		
13-15	255/264 (97)	263/277 (95)
9-12	9/264 (3)	14/277 (5)
3-8	0/264 (0)	0/277 (0)
Paresis arm	143/264 (54)	155/277 (56)
Paresis leg	151/264 (57)	161/277 (58)
Disorientation/confusion	88/264 (33)	119/277 (43)
Headache	129/264 (49)	118/277 (42)
Seizure	7/264 (3)	10/277 (4)
Dysphasia	70/264 (26)	74/277 (27)
Gait disturbance	217/264 (82)	231/277 (83)
Antithrombotic meds	134/264 (51)	135/277 (49)
Bilateral hematomas	42/264 (16)	39/277 (14)
CT image^d^		
Hypodense	168/263 (64)	187/275 (68)
Isodense	97/263 (37)	96/275 (35)
Hyperdense parts	26/263 (10)	33/275 (12)
Membranous	37/263 (14)	44/275 (16)
Maximal hematoma width, mean (SD), mm	20.3 (4.6)	21.0 (5.2)
Midline shift, mean (SD), mm	6.6 (4.1)	6.7 (4.2)
Operative variables		
Duration of surgery, mean (SD), min	40.0 (14)	39.0 (14.5)
Irrigation volume, mean (SD), mL	1717 (790)	1620 (748)
General anesthesia	150/264 (57)	166/277 (60)

Abbreviations: BT, body temperature; CT, computed tomography; RT, room temperature.

^a^ Total numbers less than 277 for the RT group or less than 264 for the BT group indicate missing data.

^b^ Patients often presented with more than 1 symptom.

^c^ Glasgow Coma Scale scores range from 3 to 15, where the higher scores correlate with a better clinical status.

^d^ Combinations of different CT appearances are possible.

A total of 219 of 264 patients (83%) in the BT group and 235 of 277 patients (85%) in the RT group had a CT scan of the brain performed at 2 months after surgery. If the patient developed symptoms consistent with a recurrent or residual cSDH, CT was done earlier than the planned 2-month follow-up visit. Overall, 118 of 219 patients (54%) in the BT group and 148 of 235 patients (63%) in the RT group had some residual hematoma on their CT. Most residual hematomas detected by CT at the planned control at 2 months were small and without clinical significance. As expected, symptomatic hematomas requiring reoperation were larger (mean [SD] size, 19.1 [2.7] mm in the BT group; 18.3 [3.2] mm in the RT group) compared with the asymptomatic, nonoperated hematomas (mean [SD] size, 7.6 [4.6] mm in the BT group; 7.5 [5.1] mm in the RT group).

### Primary End Point

**Figure 2.**
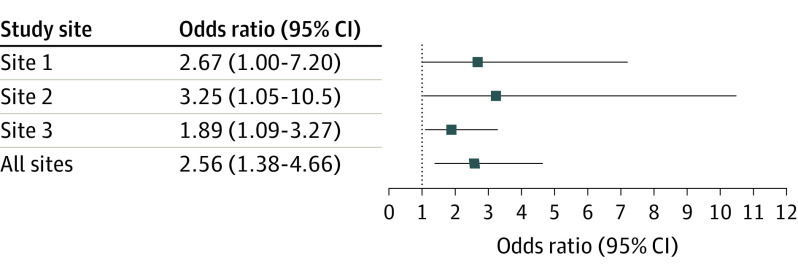
Odds Ratios and 95% CIs of Postoperative Recurrence of Chronic Subdural Hematoma for Irrigation at Room Temperature Compared With Body Temperature Data are shown for each study center, as well for all sites combined.

In the RT group, there were 39 of 277 patients (14%) with recurrence requiring reoperation compared with 16 of 264 patients (6%) with recurrence in the BT group (odds ratio [OR], 2.56; 95% CI, 1.38-4.66; *P* < .001). In an intention-to-treat analysis, 15 of 19 patients (79%) that were excluded immediately (9 in the BT group; 6 in the RT group) (Figure 1) could be analyzed. The remaining 4 randomization envelopes were discarded due to erroneous double randomization of 4 patients with bilateral hematomas and were therefore also excluded from the intention-to-treat analysis. This intention-to-treat analysis confirmed that significantly more recurrences were seen in the RT group (OR, 2.57; 95% CI, 1.40-4.71; *P* < .001). Of the 39 recurrences in the RT group, 30 (79%) occurred within the first 2 months compared with 12 of 16 (75%) in the BT group. If imputing the 5 patients lost to follow-up as having recurrences, the difference between groups was still significant. Figure 2 shows the effect across the participating study sites presented as ORs together with 95% CIs for each site, as well as in combination. This demonstrates that results were consistently in favor of irrigation fluid at body temperature across all sites.

### Secondary End Points

Mortality was 20 of 277 (7%) in the RT group compared with 14 of 264 (5%) in the BT group (OR, 1.38; 95% CI, 0.75-2.55; *P* = .27). Complication frequency was 25 of 277 (9%) in the RT group compared with 20 of 264 (7.5%) in the BT group (OR, 1.2; 95% CI, 0.66-2.2; *P* = .39). A detailed complication profile for each group is available in eTable 2 in Supplement 2. Quality of life measured by EQ-5D-3L at 6 months showed no significant difference between groups with mean (SD) index scores of 0.76 (0.21) for the BT group and 0.75 (0.24) for the RT group (*P* = .48). The index score for the Swedish normal population within the same age group is 0.74 and thus, is similar to the postoperative index score of the 2 study groups.^21^

## Discussion

In this multicenter randomized clinical trial, we showed that cSDH managed with burr hole evacuation and intraoperative irrigation at body temperature (37 °C) resulted in a statistically significant and clinically relevant reduction of cSDH recurrence compared with patients treated with intraoperative irrigation at room temperature (22 °C). There were no differences in complication frequency, quality of life, or mortality between groups. These results confirm the findings of our previously published retrospective single-center study.^16^

In this randomized clinical trial, the baseline factors were comparable, except for more cases of preoperative confusion/disorientation in the RT group compared with the BT group. However, it is unlikely that this had an impact on the results because bilateral hematomas, hematoma size, and mass effect (midline shift) did not differ between the 2 groups.

One of the reasons why irrigation of body temperature is superior to irrigation at room temperature in the evacuation of cSDH might be that rinsing the subdural space from factors mediating hematoma progression is more effective. It has been shown that the aqueous solubility of organic materials is doubled with every 20 °C increase of temperature.^13^ Furthermore, using irrigation fluid at room temperature might have a negative effect on coagulation compared with irrigation fluid at body temperature.^14^

The efficacy of postoperative drainage after cSDH evacuation has been established with solid evidence, but the use of other adjuncts is either not backed by evidence or has failed to demonstrate net benefit. Many randomized clinical trials exploring different aspects of cSDH surgery are ongoing.^22,23^ Corticosteroid use has been shown to reduce recurrence but at the cost of more adverse events.^24^ Trials of atorvastatin have not yet provided convincing evidence to be of benefit in cSDH surgery.^25^ Recently, there has been an interest in both tranexamic acid and embolization of the middle meningeal artery, and we are awaiting results from ongoing trials to evaluate their efficacy.^23^ Such adjuncts may not only reduce recurrences after cSDH surgery but may also be initiated in patients with smaller cSDH to reduce the need of repeated surgeries. Our study, however, provides a refinement of the current surgical practice and can be easily implemented, without any patient-related contraindications or increased risk.

### Strengths and Limitations

A strength of this study was that it was conducted as a multicenter randomized clinical trial with a follow-up period of 6 months. Furthermore, the 3 participating neurosurgical departments each have defined population-based catchment areas, covering almost 60% of the Swedish population. All departments used the same surgical technique, and the benefit of body temperature irrigation was observed at all participating sites. Digitalized medical records used at the neurosurgical departments as well as the local hospitals within the catchment areas facilitated a complete follow-up of all but 5 study participants.

A main limitation of this study was the inability to blind the treating surgeon to the irrigation fluid temperature. However, no significant difference was detected in the mean operating time or the amount of irrigation used between the 2 groups, indicating an unchanged surgical practice. Even though all departments use the same surgical technique, subtle differences cannot be ruled out. Another limitation is that of the 600 randomization allocations prepared, a total of 541 study participants had a complete follow-up. Unfortunately, 30 randomization envelopes were lost at 1 of the study sites and thus could not be used for randomization. The treatment allocations for these 30 envelopes were equally distributed between the 2 study groups. However, the obtained sample size still exceeded the number of study participants needed for a power of 80%. Finally, we did not register the reasons for nonenrolment, which was nevertheless in accordance with our study protocol.^15^

## Conclusions

This was, to our knowledge, the first randomized clinical trial comparing the efficacy of irrigation fluid of different temperatures used in cSDH surgery. The results of our study demonstrate the superiority of body temperature irrigation compared with room temperature irrigation. When irrigation is used in cSDH surgery, body-tempered irrigation fluid should be considered standard of care.

## Notes

Supplement 1. Trial Protocol

Supplement 2. **eAppendix.** Investigators and Study Sites **eTable 1.** Postoperative Status for BT and RT Groups Within 24 Hours After Surgery **eTable 2.** Complication Profile for the BT and RT Groups of the SIC Study

Supplement 3. Data Sharing Statement
