# Supplementary material for: Effect of Irrigation Fluid Temperature on Recurrence in the Evacuation of Chronic Subdural Hematoma: A Randomized Clinical Trial
Source: JAMA Neurol. 2022 Nov 21;80(1):58–63. doi: 10.1001/jamaneurol.2022.4133 (PMC9679960; doi:10.1001/jamaneurol.2022.4133)
Supplement: Supplement 1. — Trial Protocol [file jamaneurol-e224133-s001.pdf]

The Swedish study of Irrigation fluid temperature in the evacuation of Chronic subdural hematoma (SIC!)

- A study protocol for a multicenter randomized controlled trial

Andreas Bartley MD, Asgeir Jakola MD PhD, Jiri Bartek Jr. MD, Jimmy Sundblom MD PhD, Marie Fält RN, Petter Förander MD PhD, Niklas Marklund MD PhD, Magnus Tisell MD PhD

Background

A chronic subdural hematoma (cSDH) consists of an accumulation of blood and degraded blood products located between the dura and the brain. cSDH often expand over time causing progressive neurological symptoms due to brain compression. Symptoms include altered mental state, hemiparesis, headache and in severe cases loss of consciousness and even death. Chronic subdural hematomas mainly affect the elderly due to brain atrophy, an increased tendency to fall and a high prevalence of antithrombotic medications.[1,2]
The incidence of cSDH in individuals over 70 years is approximately 58 per 100 000 per year.[3] This makes cSDH one of the most common conditions requiring neurosurgical treatment.

Symptomatic cSDH can in the majority of cases be treated by surgical evacuation, most often performed as surgical evacuation via one or more cranial burr holes.[4,5] Results from previous studies, including a randomized controlled trial, have showed that post-operative drainage reduces the recurrence rate.[6,7]
The surgical procedure can be combined with intraoperative irrigation of the subdural space, and although potential benefits with this technique have not yet been ascertained in a controlled manner, there is evidence in favour of intraoperative irrigation.[7,8] Still, the recurrence of cSDH is a major clinical problem with an estimated recurrence frequency of 3-21.5% with closed-system drainage %.[9]

The irrigation fluid temperature may have an impact on recurrence rates of cSDH, possibly due to negative effects on the coagulation when using fluid at room temperature. [10-12] It is also plausible that irrigation fluid at body temperature may

increase the solubility of the cSDH, thereby facilitating evacuation. The use of body vs. room temperature irrigation fluid varies between neurosurgical departments, and possibly even between individual surgeons. When a poll on the neurosurgical networking site *www.neurosurgic.com* addressed this particular question, the result of 620 respondents showed that 57 % use irrigation fluid at body temperature and 40 % irrigation fluid at room temperature and 3 % did not use any intraoperative irrigation.

The aim of this multicenter randomised controlled study is to investigate whether irrigation fluid temperature influence clinical outcome and recurrence rates in cSDH.

Hypothesis

The intraoperative use of irrigation fluid at body temperature results in lower recurrence rates when compared to irrigation fluid at room temperature. Consequently, the defined null hypothesis will be that there is no difference between the treatment groups.

Primary aim

The primary aim is to investigate whether irrigation with body-temperature fluid reduces recurrence rates of cSHD compared to room-temperature fluid.

Secondary aims

Secondary endpoints include health related quality of life, complication profile and frequency as well as mortality rate.

Methods

Study design

A multicentre randomised controlled trial (RCT) evaluating the use of irrigation fluid at body temperature, 37°C, versus irrigation fluid at room temperature in burr hole evacuation of cSDH. Except from random allocation of treatment between irrigation fluid temperatures the management of the participants will not differ from the current management of patients treated for cSDH. A 1:1 block randomization will be performed at each clinic.

Participating sites are the neurosurgical departments at Sahlgrenska University Hospital (Gothenburg, Sweden), Karolinska University Hospital (Stockholm, Sweden) and the Uppsala University Hospital (Uppsala, Sweden).

The primary endpoint of the study is the recurrence rate within a 6-month follow up period. Secondary endpoints include mortality rate, complication profile and frequency and health related quality of life at follow-up.

The study is registered in *clinicaltrials.gov* and reported according to the SPIRIT guidelines for reporting a RCT study protocol.[13]
Trials registration: ClinicalTrials.gov, NCT02757235. Registered 05/02/2016.

Study participants

Patients diagnosed with cSDH, where surgical evacuation is indicated, will be screened for inclusion in the study.

Inclusion criteria

- Patients with cSDH requiring burr hole evacuation
- Patients older than 18 years of age.

Exclusion criteria

- cSDH requiring surgical treatment other than burr hole evacuation.
- cSDH in a patient with an intracranial arachnoidal cyst.
- cSDH in a patient with a CSF-shunt.
- Patients who have undergone intracranial surgery before.

Patients with bilateral cSDH will be treated with the same irrigation fluid modality on both sides, and analysed as a single study participant.

Informed consent

Informed consent will be obtained by the attending neurosurgeon prior to surgery of the cSDH. If the patient is unable to give consent, consent will be sought from a close relative. Withdrawal from the study is possible at any time, in accordance with the latest version of the declaration of Helsinki 2013.[14]

Baseline variables

Variables will be documented in the case report form (CRF) preoperatively, intraoperatively and within 24 hours postoperatively by the attending neurosurgeon.

Preoperative variables:

- Age, sex, limb weakness, Glasgow Coma Scale (GCS), disorientation, seizures, dysphasia, headache, gait disturbance, mid-line shift, maximal width of the hematoma on CT and presence of uni- or bilateral cSDH. Concomitant medication will also be recorded for aspirin, clopidogrel, warfarin and new oral anticoagulants (NOACs). Dexamethasone use will not be recorded since it is not used in the treatment of cSDH in Sweden.

Intraoperative variables:

- Unilateral or bilateral surgery, general or local anaesthesia, duration of surgery, irrigation fluid volume

Postoperative variables (within 24 hours)

- GCS, limb weakness, disorientation, dysphasia, headache, convulsions, gait disturbance, duration of postoperative drainage

Surgical technique

Current management of cSDH at all participating departments is burr hole evacuation with intraoperative irrigation followed by active subgaleal drainage as described by Gazerri et al.[15]

The patient will undergo surgery in the supine position under general or local anaesthesia, and 1-2 burr holes are placed over the maximum width of the hematoma. The dura is opened in a cruciate fashion and coagulated by bipolar diathermia. The subdural space is irrigated with Ringer´s lactate of either body- or room temperature according to the result of the randomization. For irrigation a 50 ml syringe and a soft catheter is used. When the irrigation fluid runs clear a subgaleal drainage is inserted over the burr hole and tunnelled away from the skin incision. The drainage is connected to a collection bag with active suction ensuring continuous drainage. The patient is kept in the supine position until the drainage is removed the day after surgery. If the surgeon decides that a drain cannot be safely inserted the patient will be excluded from the study, although this should be a rare event du to the subgaleal location of the drainage.

Randomization and blinding

A total of 600 opaque envelopes (200 for each participating centres) with sequential study numbers containing randomly assigned irrigation fluid temperatures will be prepared. Importantly, the envelopes are not transparent even under direct illumination and will be kept sealed until the patient is draped in the operation room. Also, the envelopes are interconnected so that it is impossible to open these in the wrong order by mistake or on purpose. The envelope will be opened at the time of surgery by the surgeon performing the procedure.
It will not be possible mask the treatment allocation from the treating surgeon, nevertheless measures will be taken to minimise bias.

1. The patient will not be informed of treatment allocation.
2. To minimize detection and selection bias the treatment allocation will not be documented in medical records.
3. The investigator performing the statistical analyses will be blinded to treatment assignment until the final analysis is completed.

Outcome measures

1. Recurrence rate of same-sided cSDH requiring surgery within 6 months.

2. EuroQol 5D (version 3L)
EuroQol 5D-3L (EQ-5D 3L) is a generic measure of health related quality of life (HRQL).[16] The questionnaire contains 5 questions addressing mobility, self-care, usual

activities, pain/discomfort and anxiety/depression. Each question can be answered with "no problem", "slight problem" or "major problem".

3. Complications within the follow up period will be assessed using the Landriel Ibanez classification system.[17] This system grades complications in 4 grades (Grade 1: no invasive treatment required, Grade 2: invasive treatment required, but not ICU. Grade 3: invasive treatment required and ICU, Grade 4: death).

4. Mortality will be analyzed as overall survival 6 months post-treatment.

Follow up

The follow-up period is 6 months. Within this period recurrences requiring surgery are registered together with any complications and mortality rate of the participants.
At 2 months after surgery a CT-scan is performed to assess any residual hematoma.
At 6 months after surgery HRQL will be assessed using the EQ-5D questionnaire, mailed to each participant.
Any loss to follow up will be recorded.

Indication for reoperation

This is based on usual clinical practice and is consequently a decision made by the treating physician, often the neurosurgeon who is on-call. The indication for reoperation is typically based on the presence of significant residual or recurrent neurological symptoms (headache, paresis, dysphasia, etc.). If symptoms are present, a CT-scan is performed and if there is cSDH causing mass effect correlating with the symptoms the patient will undergo reoperation.

Sample size calculations

The total number of patients will be 496 (248 in each group, respectively) for a power of 80 %. The calculation is bases on a retrospective pilot study performed at Sahlgrenska University Hospital, 2013-14, with a 5 % versus 12 % recurrence rate when comparing irrigation with body-temperature fluid versus room-temperature fluid, respectively. A p-value of < 0.05 is considered statistically significant. To compensate for a loss to follow-up, we aim to include a total of 600 patients. Each centre will recruit 200 study participants ensuring homogenous recruitment between the participating centres. Although we do not anticipate a large loss to follow-up regarding the primary endpoint, we believe that this may be the case for the secondary endpoints. Thus, we will mainly compensate for a loss to follow-up regarding the secondary outcome measures such as EQ-5D 3L.

Data management and statistical analysis

A statistician blinded for treatment assignments will perform the analysis according to the intention to treat principle.

The primary endpoint (rate of recurrences requiring surgery within 6 months) will be analysed using x^2-test for frequency comparison.
Secondary endpoints will be analysed using appropriate statistics. Categorical data will be compared using x^2-test or Fishers exact test. Normally distributed numerical data will be analysed using t-test and Mann Whitney U-test if skewed.

Data will be collected the CRF and on password protected computers. CRF and consent forms are stored in locked rooms at each department. Each study participant's documents are identified by the randomization number, ensuring confidentiality.

Data monitoring

An interim analysis will be performed by an independent statistician when each site has 100 subjects with a completed 6 month follow up. The interim analysis will focus on differences in mortality and recurrence rates (the primary endpoint) between study groups. In addition, each site will cross monitor each other regarding obtained consents and source of clinical data.

Ethical approval

The study has been approved by the Regional Ethics Committee in Gothenburg, Sweden, January 19, 2016 (reference number: 932-15).

Discussion

To the best of our knowledge this is the first randomised controlled trial comparing irrigation fluid temperatures used during surgical evacuation of chronic subdural hematomas. Chronic subdural hematoma is an increasingly common condition affection the elderly, believed to be linked to demographic changes, extensive use of anticoagulants/anti-platelet therapy and easy access to radiological examination of the brain.[18] The recurrence rate is rather high, with a need for randomized controlled trials in an effort to optimize treatment strategies in hope of reducing the recurrence rates. A possible variable influencing the recurrence rate is the temperature of the irrigation fluid used during hematoma evacuation,
by affecting solubility of the hematoma and/or coagulation. To test this theory, the current study was initiated.

If a decreased recurrence rate is observed in this multicenter randomized controlled trial, a new surgical standard of irrigation temperature can be established. If the need of reoperation could be diminished this has obvious gains both for the patient and the health care system.
Strengths of the study include the RCT setup and the involvement of three independent neurosurgical centers in Sweden, covering 60 % of the Swedish population. All of these with population-based catchment areas and almost identical surgical technique. Also, a digitalized patient chart system ensures easy access facilitating follow-up. Limitations include the inability to blind the treating surgeon at to the temperature of the irrigation fluid, generating possible bias. Furthermore, although the surgical strategy is very

homogeneous among the participating centers, subtle differences in the surgical method between individual surgeons and also between the centers cannot be excluded.

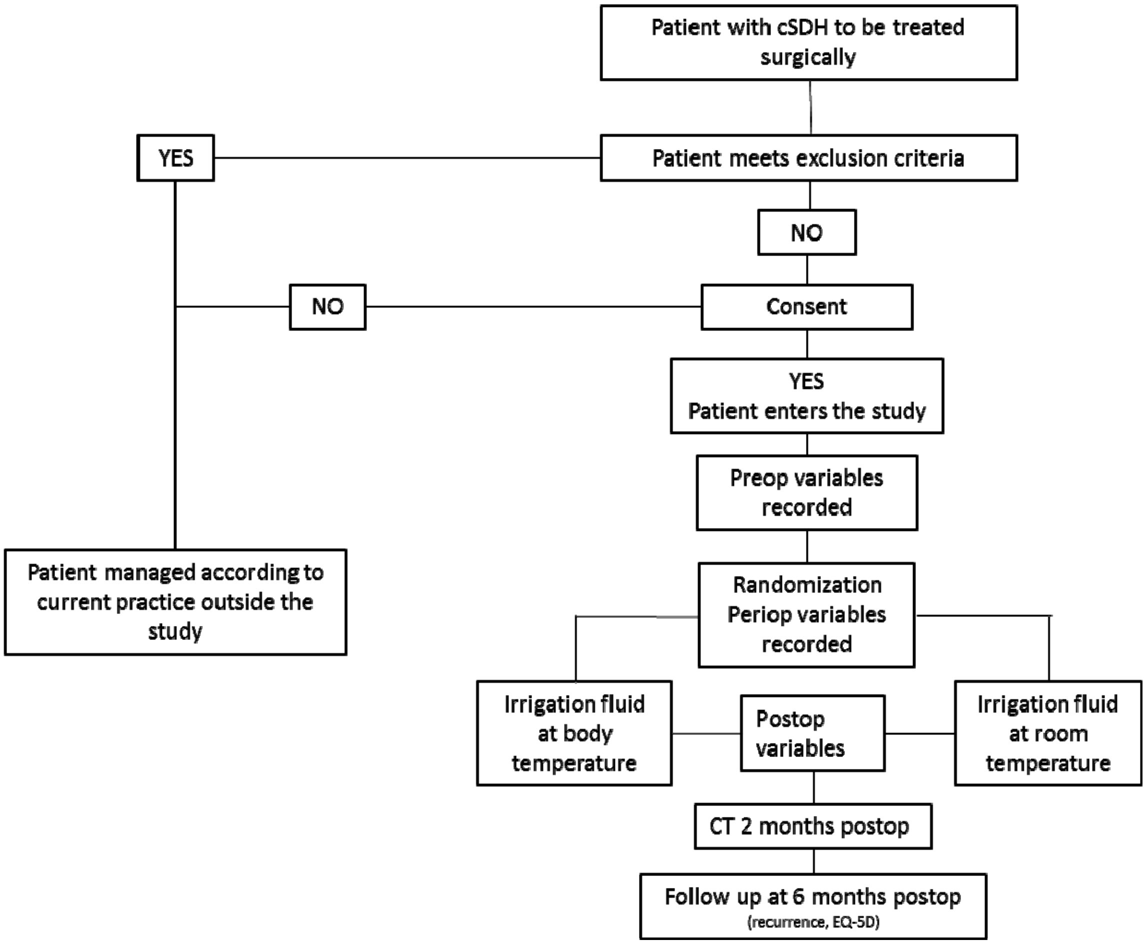

Figure 1: SIC-study flowchart

References

1. Asghar M, Adhiyaman V, Bates A, et al: Chronic subdural haematoma in the elderly – a North Wales expericence. *J R Soc Med* 2002; 95:290-292
2. Yang AI, Balser DS, Mikheev A, et al. Cerebral atrophy is associated with development of chronic subdural haematoma. *Brain Inj* 2012; 26: 1731-1736
3. Kudo H, Kuwamura K, Tamaki N, et al: Chronic subdural haemtoma in eldery people; present status on Awaji Island and epidemiological prospect. *Neurol Med Chir* 1992; 32: 207-209
4. R. Weigel, P. Schmiedek, J.K. Krauss: Outcome of contemporary surgery for chronic subdural haematoma: evidence based review. *J Neurol Neurosurg Psychiatry.* 2003; 74:937-943
5. T. Santarius, P.J. Hutchinson: Chronic subdural haematoma: time to rationalize treatment? *Br J Neurosurg.* 2004; 18:328-332

6. T. Santarius, P. Kirkpatrick, P. Hutchinson, et al: Use of drains versus no drains after burr-hole evacuation of chronic subdural haematoma: a randomized controlled trial. *Lancet*. 2009; 374: 1067-1073
7. W. Liu, N A. Bakker, R J M Groen: Chronic subdural hematoma: a systematic review and meta-analysis of surgical procedures. *J Neurosurg*. 2014; 121: 665-673
8. Henning R, Kloster R: Evacuation of chronic subdural haematomas followed by continuous inflow and outflow irrigation. *Acta Neurochir (Wien).* 2007; 141(2): 171-6
9. Yu GJ, Han CZ, Zhang M, et al. Prolonged drainage reduces the recurrence of chronic subdural hematoma. Br J Neurosurg. 2009;23(6):606
10. Watts DD, Trask A, Soeken K, et al: Hypothermic coaglulopathy in trauma: effect of varying levels of enzyme speed, platelet function and fibrinolytic activity. *J Trauma*. 1998; 44: 846-54
11. Reynolds L, Beckmann J, Kurz A: Perioperatvie complications of hypothermia. *Best Pract Res Clin Anaesthesiol.* 2008; 22: 645-57
12. Roher MJ, Natale AM. Effect of hypothermia on the coagulation cascade. *Crit Care Med.* 1992; 20: 1402-5
13. Chan A-W, Tetzlaff J M, Moher D, et al: SPIRIT 2013 explanation and elaboration: guidance for protocols of clinical trials. *BMJ.* 2013; 346:e7586
14. World Medical Association Declaration of Helsinki Ethical Principles for Medical Research Involving Human Subjects, *JAMA.* 2013; 310(20):2191-94
15. Gazzeri R, Galarza M, Eposito S, et al, *Acta Neurochir* 2007; 149:487-493
16. The EuroQol Group. EuroQol–a new facility for the measurement of health related quality of life. *Health Policy* 1990; 16: 199–208.
17. Landriel Ibanez FA, Hem S, Carrizo A: A new classification of complications in neurosurgery. *World Neurosurg.* 2011; 75(5-6): 709-15
18. Adhiyaman V, Chattoopadyay I, Abraham S, et al. Increasing incidence of chronic subdural haematoma in the elderly. *QJM* 2017; (Epub ahead of print)

List of abbreviations

cSDH: chronic Subdural Hematoma
RCT: Randomized Controlled Trial
HRQL: Health Related Quality of Life
CRF: Case Report Form
ICU: Intensive Care Unit
GCS: Glasgow Coma Scale
SPIRIT: Standard Protocol Items: Recommendations for Interventional Trials

Declarations

Ethics approval and consent to participate

The regional ethical review board of Gothenburg, Sweden, reviewed the project and the study was approved January 19, 2016 (reference number: 932-15).
