# Supplementary material for: Effect of Irrigation Fluid Temperature on Recurrence in the Evacuation of Chronic Subdural Hematoma: A Randomized Clinical Trial
Source: JAMA Neurol. 2022 Nov 21;80(1):58–63. doi: 10.1001/jamaneurol.2022.4133 (PMC9679960; doi:10.1001/jamaneurol.2022.4133)
Supplement: Supplement 2. — eAppendix. Investigators and Study Sites eTable 1. Postoperative Status for BT and RT Groups Within 24 Hours After Surgery eTable 2. Complication Profile for the BT and RT Groups of the SIC Study [file jamaneurol-e224133-s002.pdf]

Supplementary Online Content

Bartley A, Bartek Jr J, Jakola AS, et al. Effect of irrigation fluid temperature on recurrence in the evacuation of chronic subdural hematoma: a randomized clinical trial. *JAMA Neurol*. Published online November 21, 2022. doi:10.1001/jamaneurol.2022.4133

eAppendix. Investigators and Study Sites

eTable 1. Postoperative Status for BT and RT Groups Within 24 Hours After Surgery

eTable 2. Complication Profile for the BT and RT Groups of the SIC Study

This supplementary material has been provided by the authors to give readers additional information about their work.

© 2022 Bartley A et al. *JAMA Neurology.*

eAppendix. Investigators and Study Sites

Andreas Bartley – Sahlgrenska University Hospital, Gothenburg
Asgeir S. Jakola – Sahlgrenska University Hospital, Gothenburg

Magnus Tisell – Sahlgrenska University Hospital, Gothenburg

Jiri Bartek Jr. – Karolinska University Hospital, Stockholm

Marie Fält - Karolinska University Hospital, Stockholm
Petter Förander - Karolinska University Hospital, Stockholm
Jimmy Sundblom – Academic Hospital, Uppsala
Niklas Marklund – Academic Hospital, Uppsala

© 2022 Bartley A et al. *JAMA Neurology.*

eTable 1. Postoperative Status for BT and RT Groups Within 24 Hours After Surgery

Variable	BT-group (N=264)	RT-group (N=277)	p-value
Persisting paresis - n/N (%)	30/264 (11%)	42/277 (16%)	p=0.13
- improved	20/30 (67%)	27/42(64%)	
- same	6/30 (20%)	8/42 (19%)	
- worse	4/30 (13%)	7/42 (17%)	
Persist. dysphasia – n/N (%)	16/264 (6%)	22/277 (8%)	p=0.29
- improved	9/16 (56%)	13/22 (59%)	
- same	3/16 (19%)	3/22 (14%)	
- worse	4/16 (25%)	6/22 (27%)	
Persist. Confusion – n/N (%)	50/264 (19%)	66/277 (24%)	p=0.10
- improved	21/50 (42%)	29/66 (44%)	
- same	22/50 (44%)	21/66 (32%)	
- worse	7/50 (14%)	16/66 (24%)	
GCS – median (range)	15 (15-10)	15 (15-9)	

© 2022 Bartley A et al. *JAMA Neurology.*

eTable 2. Complication Profile for the BT and RT Groups of the SIC Study

Case	BT-group	RT-group
1	Seizure postop.	Seizure postop
2	Insular infarction, permanent neurological deficit.	Urinary tract infecion
3	Pneumonia	Urinary tract infecion
4	Small ICH, no surgical intervention, no permanent deficit.	Deep venous thrombosis
5	Pneumonia	Myocardial infarction
6	Deep venous thrombosis	Wound infection, treated with antibiotics, no surgical revision
7	Small ICH, no surgical intervention, no permanent deficit.	Ischemic stroke, permanent neurological deficit
8	Severe confusion with psychosis	Wound infection, treated with antibiotics, no surgical revision
9	Seizure postop.	Ischemic stroke, permanent neurological deficit
10	Deep venous thrombosis	Ischemic stroke, permanent neurological deficit
11	Urinary tract infection	Small ICH, no surgical intervention, no permanent deficit.
12	Urinary tract infection	Acute subdural hematoma, reoperation.
13	Acute subdural hematoma, requiring reoperation	Sepsis of unknown origin.
14	Urinary tract infection	Myocardial infarction
15	Seizure postop.	Urinary tract infecion
16	Wound infection, treated with antibiotics, no revision surgery	Ischemic stroke, no permanent neurological deficit
17	Urinary tract infection	Urinary tract infecion
18	Pneumonia with sepsis, fatality.	Pneumonia
19	Acute subdural hematoma, requiring reoperation	Urinary tract infecion
20	Wound infection, treated with antibiotics, no revision surgery.	Urinary tract infecion
21		Ischemic stroke, permanent neurological deficit
22		Wound infection, treated with antibiotics, no surgical revision
23		Pneumonia
24		Renal failure
25		Seizure postop.

© 2022 Bartley A et al. *JAMA Neurology.*
