# Supplementary material for: Effect of Irrigation Fluid Temperature on Recurrence in the Evacuation of Chronic Subdural Hematoma: A Randomized Clinical Trial
Source: JAMA Neurol. 2022 Nov 21;80(1):58–63. doi: 10.1001/jamaneurol.2022.4133 (PMC9679960; doi:10.1001/jamaneurol.2022.4133)
Supplement: Supplement 3. — Data Sharing Statement [file jamaneurol-e224133-s003.pdf]

Data Sharing Statement

Bartley. Effect of Irrigation Fluid Temperature on Recurrence in the Evacuation of Chronic Subdural Hematoma. *JAMA Neurol*. Published November 21, 2022.
doi:10.1001/jamaneurol.2022.4133

Data
Data available: Yes
Data types: Deidentified participant data
How to access data: All data requests should be submitted to the corresponding author (andreas.bartley@vgregion.se) for consideration.
When available: With publication

Supporting Documents
Document types: None

Additional Information
Who can access the data: Researchers whose proposed use of the data is approved.
Types of analyses: Any purpose.
Mechanisms of data availability: after approval of a propsal.
